# Numerical Simulations and Bending Fatigue Experiments of Compensation Ropes Adopted in Highspeed Railway

**DOI:** 10.3390/ma19101983

**Published:** 2026-05-11

**Authors:** Yingxin Zhao, Qingyuan Zhao, Fengyuan Li, Haibo Zhang, Fei Du, Xiyue Yu, Aiguo Zhao

**Affiliations:** 1 Standards & Metrology Research Institute, China Academy of Railway Sciences Corporation Limited, Beijing 100000, China; yxzhao15801042226@163.com (Y.Z.); zqy729372235@163.com (Q.Z.); 2 School of Electrical Engineering, Southwest Jiaotong University, Chengdu 610000, China; lifegnyuan.1991@163.com; 3 College of Civil Engineering, Nanjing Tech University, Nanjing 211816, China; njtech_df@163.com (F.D.); 18226406495@163.com (X.Y.)

**Keywords:** steel wire rope, bending fatigue, elastic slender rod theory, equivalent diameter model

## Abstract

In high-speed train traction power supply systems, compensation ropes serve as critical transmission components to ensure system stability. These ropes are specially designed as right-hand alternating lay wire ropes. During tension compensation of the contact wire, the compensation rope undergoes repeated bending around the ratchet device, making it susceptible to fatigue fracture. This study conducted bending fatigue tests on compensation ropes with complete structural configurations in accordance with GB/T 12347-2008. The stress distribution and deformation evolution induced by bending were simulated using the finite element method, enabling fatigue life prediction under cyclic bending conditions. Given the significant convergence difficulties encountered in large-deformation bending simulations of the full structural model, this study innovatively adopts Love’s elastic thin-rod theory as an alternative approach, which avoids the computational prohibitions of full-scale helical modeling while preserving critical bending stiffness characteristics. The results demonstrate that the equivalent elastic modulus derived from Love’s elastic thin-rod theory closely matches the modulus obtained through stress–strain curve fitting from strand tensile tests. Furthermore, under identical axial tensile loads, the equivalent diameter model and the full-structure finite element model exhibit nearly identical end elongations. The predicted bending fatigue life using the equivalent diameter model agrees well with experimental results, and the fatigue fracture mechanisms are further revealed through microscopic morphology analysis, collectively confirming that the proposed equivalent modeling strategy provides an efficient and reliable solution for fatigue life prediction of complex wire rope structures under coupled tension–bending conditions.

## 1. Introduction

**Figure 1:**
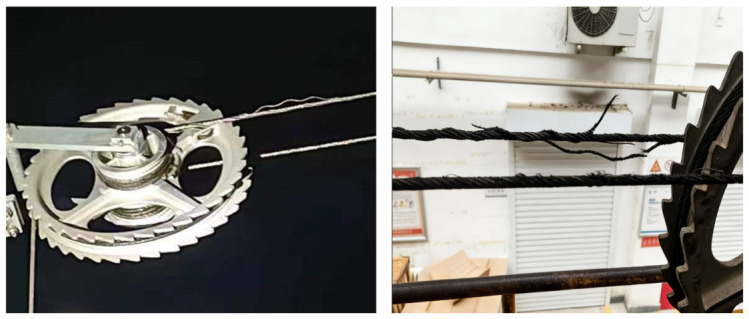
Broken-strand compensation rope.

In modern Chinese high-speed trains, electric power has replaced traditional power systems. As operating speeds continue to increase, the stability of the train power supply system is subject to increasingly stringent requirements. Serving as a transmission component in the ratchet-type compensation device, steel wire ropes play a critical role in maintaining proper tension between the train and the contact wire—hereinafter referred to as compensation ropes. During the tension compensation process between the train and the contact wire, these ropes undergo repeated bending at the ratchet wheel. Such cyclic bending can lead to fatigue fracture, potentially compromising the safe operation of high-speed trains. As shown in Figure 1, broken strands of the compensation rope were observed near the small pulley of the ratchet compensation device.

Investigating the mechanical behavior of steel wire ropes is fundamental to optimizing structural design and predicting fatigue life. Hruska [1,2,3] pioneered a mechanical model for steel wire ropes under tensile loading based on the fiber assumption, although it did not account for the effects of Poisson’s ratio, torsion, or bending stress. Building on Love’s generalized curved rod theory [4], Phillips et al. [5] and Costello [6] extended this work by incorporating these effects, enabling a more comprehensive analysis of contact behavior and static mechanical response in steel wire ropes. Raoof et al. [7,8] employed a semi-continuous analytical method to study multilayer wire strands and characterized the inter-wire contact, bending behavior, and fatigue performance. Jiang et al. [9] proposed a precise finite element modeling approach for simple straight-strand ropes, achieving strong agreement with Costello’s theoretical predictions and Utting’s experimental results, and enabling effective investigation of highly nonlinear local phenomena such as contact stress, residual stress, and plastic deformation. Usabiaga et al. [10] developed a mechanical model for steel wire ropes under combined tension and torsion, based on beam theory and Love’s slender rod assumptions, revealing that stress variations in the outer-layer wires become more pronounced under torsional loading. Moradi et al. [11] and Fontanari et al. [12,13] conducted finite element analyses on geometrically complex Warrington-Seale ropes, providing insights into failure mechanisms based on stress and deformation behavior. Vukelic et al. [14] integrated finite element analysis with Goodman’s fatigue theory to estimate the remaining fatigue life of steel wire ropes. Ahmad et al. [15] used the finite element method to investigate radial and axial stress and displacement distributions in single-strand ropes under both frictional and frictionless conditions. Their results showed larger relative displacements in the absence of friction, while relative motion was confined to contact region boundaries when friction was considered. Bruski [16] investigated the bending characteristics of steel wire ropes used in highway cable barriers and established a three-dimensional nonlinear numerical model using solid elements, achieving good agreement with experimental data. Hroncek et al. [17] simplified complex numerical models of steel wire ropes using beam elements, obtaining results consistent with experimentally measured load capacities for single-strand ropes, and subsequently extended the methodology to 6 × 7-WSC multi-strand configurations.

Hobbs et al. [18] conducted large-scale axial and bending fatigue tests on typical stay cables and suspension bridge hangers, systematically analyzing their fatigue performance and failure characteristics. Beretta et al. [19] proposed a fatigue strength prediction model for steel wires, which was successfully applied to estimate the fatigue strength of two distinct types of steel wire ropes. Giglio et al. [20,21] developed a fatigue life prediction method based on the Juvinall approach derived from S-N curves, and validated its effectiveness for bending fatigue life estimation through comparisons between simulated and experimental results. Urchegui et al. [22] performed bending fatigue tests on steel wire ropes and observed wear morphologies at wire contact regions, subsequently analyzing the influence of wear damage on fatigue life using linear elastic fracture mechanics. Argayov et al. [23] conducted a detailed analysis of wear evolution under cyclic bending loads and discussed its implications for fatigue life estimation. Winkler et al. [24] established a preliminary fatigue life model through a series of bending fatigue tests, emphasizing the role of local bending stresses. Kim et al. [25] correlated the number of bending fatigue cycles with the fracture strength of steel wire ropes, revealing that fracture strength decreases at an accelerating rate as bending cycles increase. Zhang et al. [26] investigated the bending fatigue behavior and failure mechanisms of steel wire ropes using both nondestructive quantitative inspection and manual inspection methods. Their results indicate that wear-induced fracture predominated in ropes running over steel sheaves, whereas fatigue fracture was more common in ropes running over nylon sheaves. Varney et al. [27] conducted fatigue tests on mooring wire ropes and established a life prediction model based on a progressive damage approach, with predictive accuracy confirmed by experimental data.

Guo et al. [28] evaluated the fatigue characteristics of steel wires using various analytical models and found that theoretical predictions closely matched bending fatigue test results. Winkler et al. [29] proposed a digital image-based method to measure local inter-wire deformations under bending loads and investigated fretting fatigue behavior in prestressed steel wire ropes. Peterka et al. [30] employed nondestructive testing equipment to periodically monitor damage progression in elevator wire ropes, developing a life prediction model based on observed damage indicator trends. Zhao et al. [31] predicted the bending fatigue life of steel wire ropes based on stress field intensity theory and validated the method experimentally. Zhang et al. [32] investigated the influence of broken wire distribution on bending fatigue behavior, showing that surface broken wires significantly reduce fatigue life by increasing inner wire stress and promoting localized wear. Tijani et al. [33] conducted accelerated life tests on steel wire ropes, deriving relevant parameters from observational data and subsequently using them to predict fatigue life. Wang et al. [34,35] performed bending fatigue tests under constant- and variable-amplitude loading using a custom-built test rig, finding that fracture occurred later under variable-amplitude loading, although severe core strand damage was observed. Erena et al. [36] experimentally studied the bending fatigue behavior of single-strand ropes under combined axial and bending loads. Shen et al. [37] investigated fatigue failure under combined axial and bending loading, proposed a novel test apparatus to avoid clamping-induced failure, and obtained fatigue life prediction curves. Tang et al. [38] predicted the fatigue life of helical steel wires in bridge cables under combined tension–bending cyclic loading based on linear elastic fracture mechanics, achieving good agreement between theoretical predictions and experimental results.

The research on the bending fatigue failure of high-speed railway compensating ropes, a special structural steel wire rope, is still unclear. Existing theoretical models and numerical methods struggle to effectively balance computational accuracy and engineering feasibility. Therefore, based on the Love’s theory of elastic thin rods, this paper proposes an equivalent diameter modeling method for finite element simulations of stress distribution and deformation evolution during the bending process of compensating ropes around ratchet wheels. Furthermore, by combining the finite element calculation results with classical fatigue life empirical formulas, the fatigue life under tensile–bending coupled loading conditions is predicted. By comparing the model-predicted lifespan with the experimentally measured bending fatigue lifespan of compensating ropes, the error is controlled within a reasonable range, thus verifying the effectiveness and engineering applicability of the proposed method. Therefore, the research method in this paper can be further extended to assess the fatigue lifespan of compensating ropes under different diameter ratchet wheel bending conditions. It can also be extended to predict the remaining fatigue lifespan of compensating ropes with radial defects such as untwisting, broken wires, and broken strands. This provides a theoretical basis for the routine inspection, maintenance, and lifespan management of high-speed railway contact system compensating devices, significantly reducing the time and economic costs associated with traditionally relying on extensive experiments to obtain service life.

## 2. Experimental Materials and Methods

### 2.1. Experimental Materials

**Figure 2:**
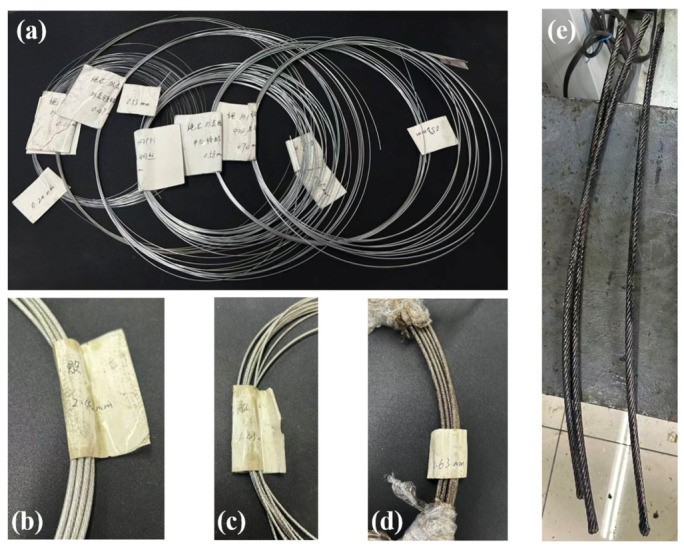
Experimental materials. (**a**) High-carbon galvanized steel wires and stainless steel wires with different diameters. (**b**) Core strand with left-lay structure and nominal diameter of 2.42 mm. (**c**) Inner strand with right-lay structure and nominal diameter of 1.43 mm. (**d**) Outer strand with left-lay structure and nominal diameter of 2.63 mm. (**e**) Whole compensation rope with right Lang’s lay structure and nominal diameter of 10.9 mm.

The high-carbon galvanized steel wires and stainless steel wires employed in this study were supplied by Jiangyin Faersheng Metal Products Co., Ltd. (Wuxi, Jiangsu Province, China). As shown in Figure 2a, high-carbon galvanized steel wires with diameters of 0.20 mm, 0.43 mm, 0.47 mm, 0.53 mm, 0.55 mm, 0.57 mm, and 0.74 mm, as well as stainless steel wires with a diameter of 0.46 mm, were selected. For each wire diameter, four groups of specimens were prepared for tensile testing. The length of each specimen was set to 500 mm. As shown in Figure 2b–d, the specimens included a core strand with a nominal diameter of 2.42 mm and a left-hand lay, an inner-layer strand with a diameter of 1.43 mm and a right-hand lay, and an outer-layer strand with a diameter of 2.63 mm and a left-hand lay. Three groups of specimens were prepared for tensile tests, and the length of each specimen was 1000 mm. As shown in Figure 2e, three specimens of a compensation rope with a nominal diameter of 10.9 mm and a right-hand alternating lay structure were prepared for tensile testing, each with a length of 350 mm. The chemical compositions of high-carbon galvanized steel wire and stainless steel wire are shown in Table 1.

### 2.2. Experimental Methods and Results

#### 2.2.1. Tensile Test of Steel Wires with Different Diameters

**Figure 3:**
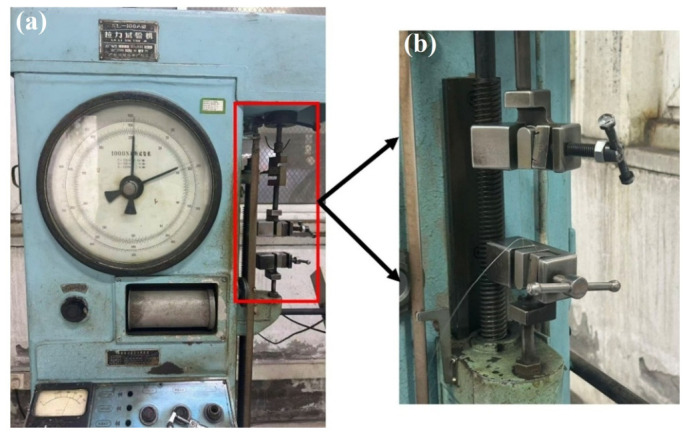
XL-100A tensile testing machine. (**a**) Overall of XL-100A tensile testing machine, for wires < 0.5 mm, max tension 1 kN. (**b**) Fixture details of the machine, steel wire wound and fastened for tensile clamping.

**Figure 4:**
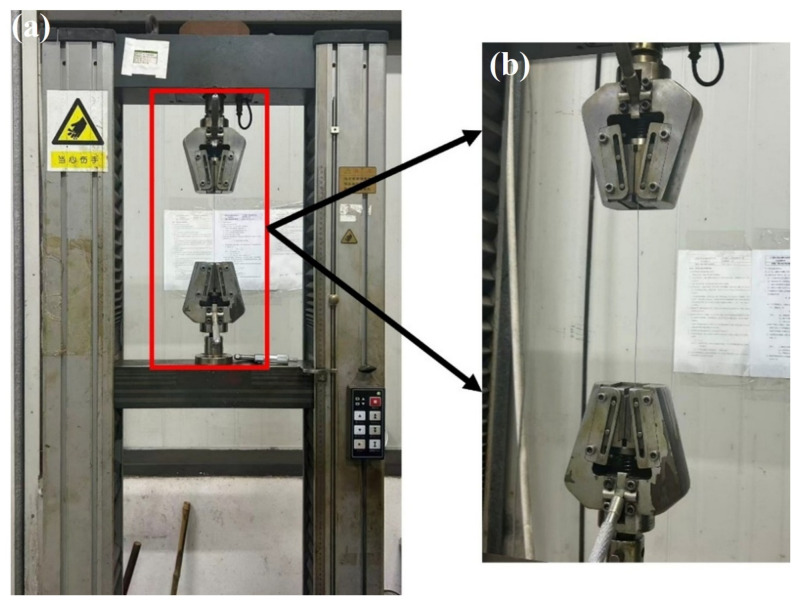
CMT4104 computer-controlled electronic universal testing machine. (**a**) CMT4104 universal tester (overall), for wires > 0.5 mm, max tension 10 kN. (**b**) Fixture details, clamp wire ends directly for tensile testing.

The tensile testing machine shown in Figure 3 is an XL-100A (Younaite, Jinan, China) model with a maximum load capacity of 1 kN, which is suitable for steel wires with diameters below 0.5 mm. During testing, the steel wire was wrapped once around the bending grips at both the upper and lower ends, and then tightened using side screws to ensure secure clamping before tension was applied. The testing machine shown in Figure 4 is a CMT4104 computer-controlled electronic universal testing machine (MTS Systems Corporation, Eden Prairie, MN, USA), with a maximum load capacity of 10 kN and a power rating of 0.4 kW, which is suitable for steel wires with diameters ranging from 0.5 mm to 1.0 mm. The steel wire was clamped directly by the upper and lower grips for tensile testing. All experiments were conducted at room temperature (20 °C) with a tensile loading rate of 5 mm/min. Before beginning the wire tensile test, remove any specimens exhibiting obvious plastic bending or permanent deformation, and apply a pre-tension equal to 1% of the wire’s breaking tensile strength in accordance with ISO 6892-1 [39] and GB/T 228.1 [40]. The primary purpose is to avoid introducing plastic deformation or work hardening during the pre-tensioning process that could affect subsequent test results. During the application of pre-tension, the load should be gradually increased until the wire is visually confirmed to be essentially straight. To ensure the accuracy of the final results, experimental data with relatively large deviations were excluded, and the remaining data were averaged for analysis.

The results of the steel wire tensile tests are presented in Table 2. The high-carbon galvanized steel wire with a diameter of 0.74 mm has a maximum average tensile strength of 789 N, indicating the greatest load-carrying capacity, whereas the high-carbon galvanized steel wire with a diameter of 0.20 mm showed the lowest breaking force of 69 N. Overall, the breaking force increased progressively with increasing wire diameter. This is because thicker wires have a larger cross-sectional area and, under the same material strength, can naturally withstand higher tensile forces. Therefore, in practical applications, steel wires with larger diameters are more suitable for regions subjected to primary loading. The maximum tensile strength was obtained for the high-carbon galvanized steel wire with a diameter of 0.53 mm, reaching 2209.8 MPa, while the minimum tensile strength was observed for the largest-diameter high-carbon galvanized steel wire (0.74 mm), with a value of 1834.3 MPa. This phenomenon can be attributed to the strain hardening effect during cold drawing. Smaller-diameter wires undergo a greater total reduction in cross-sectional area during the drawing process, resulting in higher dislocation density and more pronounced strain hardening, which leads to higher tensile strength. In contrast, larger-diameter wires experience less cumulative plastic deformation and thus exhibit lower tensile strength. The measured tensile strengths of wires of various diameters serve, on the one hand, as material parameter inputs for subsequent finite element simulations to establish finite element models of the compensation rope and its internal strands; and on the other hand, as input parameters for the fatigue strength coefficient in the fatigue life prediction model.

#### 2.2.2. Tensile Tests of Different Types of Rope

**Figure 5:**
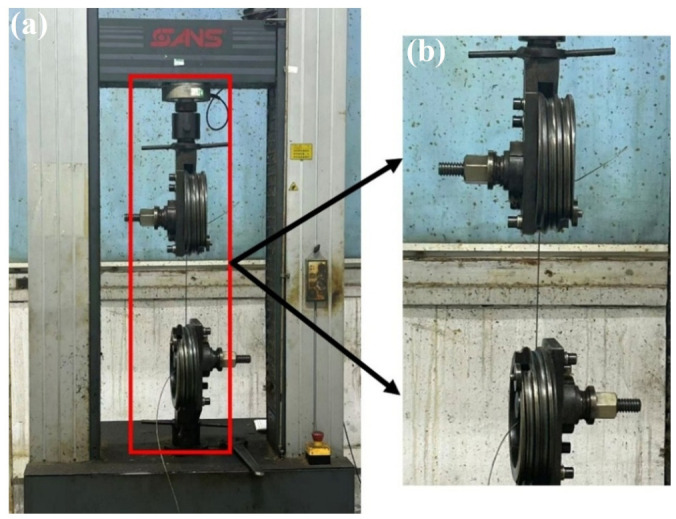
(**a**) CMT5305 universal tester (overall), for strand tension, max tension 300 kN, 2.9 kW. (**b**) Wheel fixture details, strand wound 4 turns and fastened to prevent slipping.

**Figure 6:**
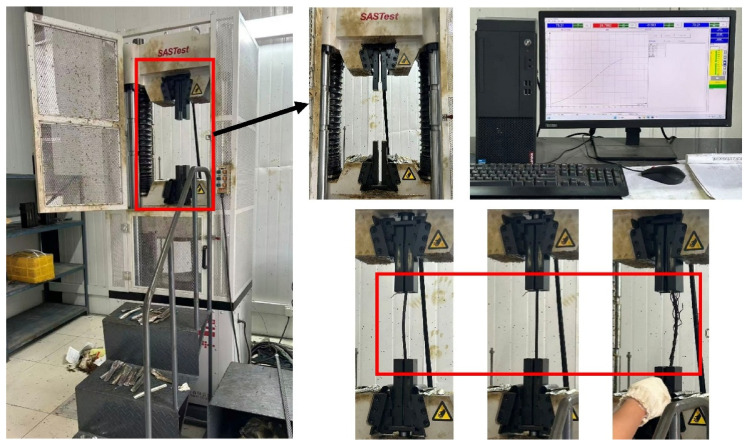
SHT4605 computer-controlled electro-hydraulic servo universal testing machine.

As shown in Figure 5, the CMT5305 (MTS Systems Corporation, Eden Prairie, MN, USA) computer-controlled electronic universal testing machine, with a maximum load capacity of 300 kN and a rated power of 2.9 kW, was used for tensile testing of single-strand structures, including the core strand, inner-layer strand, and outer-layer strand. During the tests, a specified length of the steel wire rope was wound four turns around the upper and lower drums, after which the side screws were tightened to ensure that no slippage of the strand occurred during the experiment. As shown in Figure 6, the SHT4605 (MTS Systems Corporation, Eden Prairie, MN, USA) computer-controlled electro-hydraulic servo universal testing machine, with a maximum load capacity of 600 kN and a rated power of 4 kW, was primarily used for axial tensile testing of the complete compensation rope. Special aluminum sheet molds with customized grooves were first clamped at both ends of the compensation rope, which effectively ensured stability and prevented slippage during the tensile process. The specimens were then mounted in the grips of the testing machine for loading. The lower-right corner of the figure illustrates three stages of the compensation rope tensile test: the initial loading stage, the loading progression, and final fracture.

**Figure 7:**
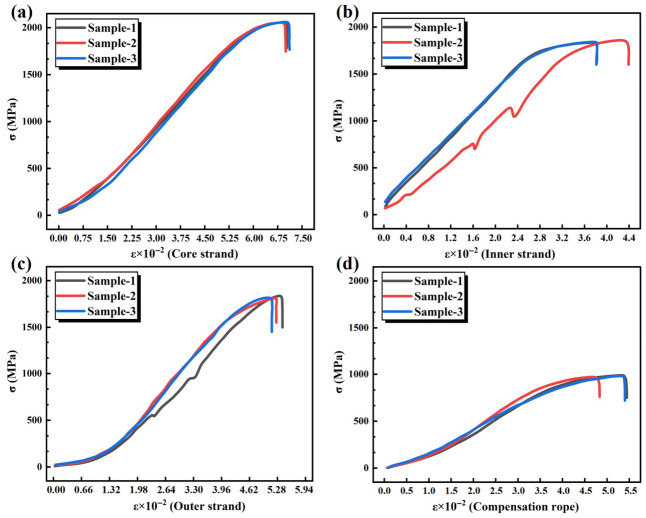
Stress–strain curves of the wires at different locations. (**a**) True stress–strain curve of core strand. (**b**) True stress–strain curve of inner strand. (**c**) True stress–strain curve of outer strand. (**d**) True stress–strain curve of whole compensation rope.

The engineering stress $\sigma_{n}$, was obtained by dividing the measured tensile force by the original cross-sectional area of the specimen. The engineering strain $\varepsilon_{n}$, was calculated by dividing the elongation by the gauge length of the extensometer. The true stress and true strain were subsequently determined using the relationships ${\sigma =\sigma }_{n}\left(1+\varepsilon_{n}\right)$ and $\varepsilon =\ln\left(1+\varepsilon_{n}\right)$. As shown in Figure 7, the stress–strain curves of the core strand, inner-layer strand, outer-layer strand, and the compensation rope are presented sequentially. For the inner-layer strand, one set of experimental data exhibited slippage, resulting in a significant deviation from the other two datasets; therefore, this dataset was excluded from further analysis. At the initial stage of loading, the strands were not fully tensioned. As the strain increased, the stress increased gradually. With continued tensile loading, the strands became fully tensioned. In this stage, the stress increased rapidly and exhibited a linear growth trend with increasing strain, which can be regarded as the elastic stage. When the strain reached a certain level, the rate of stress increase decreased, indicating the onset of the plastic stage. Upon further increase in strain, sudden fracture of the strand occurred, and the stress dropped sharply to zero. By fitting the stress–strain curves of each strand in the elastic stage shown in Figure 7, the equivalent elastic moduli of the core strand, inner-layer strand, outer-layer strand, and the compensation rope were determined to be 44.44 GPa, 62.5 GPa, 50.51 GPa, and 25.1 GPa, respectively. The core strands, inner strands, and outer strands exhibit relatively high equivalent elastic moduli when tested individually, whereas the equivalent elastic modulus of the entire compensation rope is significantly lower. This discrepancy is not due to data anomalies but is determined by the rope’s helical structure. When the individual strands are twisted into a complete rope, the elongation of the entire rope under axial tensile load is not simply the sum of the material strains of the individual strands. This is because, when the rope is under tension, the helix angle decreases and the rope diameter contracts. These geometric rearrangements generate additional axial deformation, causing the total elongation of the complete rope under the same tensile force to exceed the sum of the elongations of the individual strands when stretched separately. Consequently, the equivalent elastic modulus of the complete rope is lower than that of the individual strands. This is an inherent characteristic of the helical wire rope structure, not a testing error.

#### 2.2.3. Compensation Rope Bending Fatigue Test

**Figure 8:**
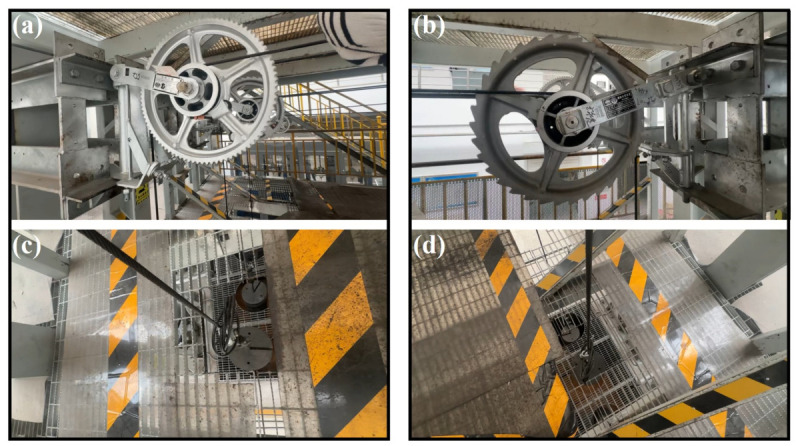
Compensating rope bending fatigue device. (**a**) The overall structure of the test device including frame, driving system, ratchet mechanism and loading system, (**b**) the ratchet assembly consisting of 180 mm small wheel and 566 mm large wheel for bending loading of the compensation rope, (**c**) the clamping and winding position of the compensation rope to ensure stable and uniform force, and (**d**) the loading and driving part providing constant tension and cyclic drive by weight and cylinder.

The bending fatigue tests of the compensation rope were conducted in accordance with the Chinese railway industry standard TB/T 2075.13–2020 [41]. The fatigue test apparatus is illustrated in Figure 8. The compensation rope was symmetrically wound around grooved sheaves with a diameter of 180 mm on both sides of the ratchet mechanism, with the same number of turns applied on each side. A counterweight with a mass corresponding to 30 kN was suspended on a large pulley with a diameter of 566 mm, and the load was jointly borne by the rope segments on both sides. The opposite end of the system was driven by a hydraulic cylinder to rotate the wheel disk, thereby inducing vertical motion of the counterweight under the action of the compensation rope. The test frequency was set to 3 cycles/min. This configuration was designed to simulate the actual service condition of the compensation rope subjected to coupled tension–bending fatigue over the small sheaves.

## 3. Theoretical Model of Compensation Ropes

### 3.1. Elastic Slender Rod Theory

**Figure 9:**
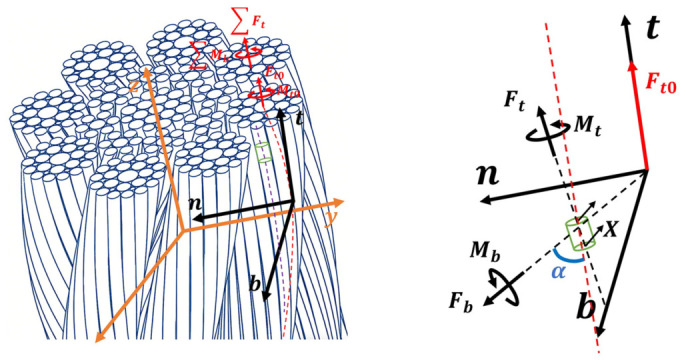
Schematic diagram of the force state of the compensation rope.

Figure 9 illustrates a small segment of an outer-layer wire of the compensation rope subjected to external forces. According to Love’s [4] elastic slender rod theory, the mechanical behavior of the wire can be expressed by Equation (1):

(1) $$\left\{\begin{array}{l} \frac{dF_{n}}{dx}-F_{n}\bar{\tau }+F_{t}\bar{k}-\bar{X}=0\\ \frac{dM_{n}}{dx}-M_{b}\bar{\tau }+M_{t}\bar{k}-F_{b}-K=0 \end{array}\right.$$

where $F_{n}$, $F_{b}$, and $F_{t}$ represent the normal, secondary normal, and tangential forces acting on the wire, respectively. $M_{n}$, $M_{b}$, and $M_{t}$ denote the normal, secondary normal, and tangential moments applied to the wire, respectively. $\bar{k}$ and $\bar{\tau }$ denote the curvature and torsion of the helical axis after loading, respectively. X is the externally applied normal force. K is the externally applied normal moment, and x is the displacement of the wire.

Assuming that the deformation of the compensation rope is uniform before and after loading, that the force is independent of position, and that K=0, Equation (1) can be simplified to Equation (2):

(2) $$\left\{\begin{array}{c} X=-F_{n}\bar{\tau }+F_{t}\bar{k}\\ F_{b}=-M_{b}\bar{\tau }+M_{t}\bar{k} \end{array}\right.$$

According to the constitutive relationship modified by Ramsey [42], $M_{b}$ and $M_{t}$ can be expressed as in Equation (3):

(3) $$\left\{\begin{array}{c} M_{b}=EI\left(\Delta k+k\xi \right)=\pi Ed^{4}\frac{\left(\Delta k+k\xi \right)}{64}\\ M_{t}=GJ\left(\Delta \tau +\tau \xi \right)=\pi Ed^{4}\frac{\left(\Delta k+k\xi \right)}{64\left(1+v\right)} \end{array}\;\right.$$

Here, E denotes the elastic modulus and G represents the shear modulus. $∆k=\bar{k}-k$ and $∆\tau =\bar{\tau }-\tau$ correspond to the variations in curvature and torsion of the helical axis before and after loading, respectively. d is the diameter of the outer wire, v is the Poisson’s ratio of the outer wire, and ξ denotes the axial strain of the outer wire along the helical axis.

By combining Equations (2) and (3), the following expression can be obtained:

(4) $$F_{b}=-EI\left(\Delta k+k\xi \right)\bar{\tau }+GJ\left(\Delta \tau +\tau \xi \right)\bar{k}$$

(5) $$X=-F_{b}\bar{\tau }+EA\xi \bar{k}$$

As illustrated in Figure 9, the axial tension in the outer-layer strand of the compensation rope is the sum of the tensions in the individual wires, while the torsional moment is the sum of the moments on each wire. Therefore, after loading, the tension and torsion in the outer-layer strand can be expressed by Equations (6) and (7):

(6) $$\sum F_{t}=F_{t0}+m\left(F_{t}sin\bar{\alpha }+F_{b}cos\bar{\alpha }\right)$$

(7) $$\sum M_{b}=M_{b0}+m\left[\left(M_{t}sin\bar{\alpha }+M_{b}cos\bar{\alpha }\right)+\bar{r}\left(F_{b}sin\bar{\alpha }-F_{t}cos\bar{\alpha }\right)\right]$$

Here, $F_{t0}=E_{0}A_{0}\varepsilon$, where $E_{0}A_{0}$ represents the axial tensile stiffness of the core wire, and ε denotes the axial strain of the core wire along the helical axis. The torsional moment is expressed as $M_{b0}=G_{0}J_{0}\varphi$, where $G_{0}J_{0}$ is the torsional stiffness of the core wire, and φ denotes the twist angle per unit length of the outer wire with respect to the core wire. α and $\bar{\alpha }$ represent the helix angles of the outer wire before and after loading, respectively. *m* represents the number of wires in the outer strand, and *r* represents the helical radius of the centerline of the wires in the outer strand.

If the complex helical structure inside the compensation rope is neglected, an equivalent approach can be adopted by treating each strand as a single wire of homogeneous material, with a diameter equal to the minimal circumscribed circle of the strand cross-section, and assuming that the wire undergoes the same axial deformation as the strand. Under this assumption, the axial tension and torsion in the strand are expressed by Equations (8) and (9):

(8) $$\sum F_{t}=E_{s}A^{\prime }\varepsilon$$

(9) $$\sum M_{b}=G_{s}J^{\prime }\varphi$$

where $E_{s}$ and $G_{s}$ are the equivalent elastic modulus and equivalent shear modulus of the strand, respectively.

By solving Equations (6)–(9) simultaneously, the following relationships are obtained:

(10) $$\begin{array}{l} E_{s}={\left(\frac{d_{0}}{D}\right)}^{2}E_{0}+m{\left(\frac{d}{D}\right)}^{2}Esin\alpha -mE{\left(\frac{d}{D}\right)}^{2}\left(1-\rho \right)\left(1-\gamma \right)\\ \left[\begin{array}{l} \left(1-\rho \right)\left(1-\gamma \right)\left(1+v\right)+{\left(1+\xi \right)}^{2}\left(\xi -1\right)\left(1+v\right)-\\ \left(1+\varepsilon \right)\left(1-\gamma \right)-{\left(1+\xi \right)}^{2}\left(\xi -1\right) \end{array}\right]\\ \frac{sin\alpha cos^{4}\alpha }{\left[16{\left(1+\xi \right)}^{3}\left(1+v\right)\right]} \end{array}$$

(11) $$\begin{array}{l} G_{s}={\left(\frac{d_{0}}{D}\right)}^{4}G_{0}+{\left(\frac{d}{D}\right)}^{4}aGsin^{2}\alpha +\left(\frac{d^{4}}{2D^{4}}\right)bEsin\alpha cos^{2}\alpha \\ -\left(\frac{8d^{2}}{D^{4}}\right)cEr^{2}sin\alpha \end{array}$$

where the coefficients a, b, and c are defined by Equation (12)–(14):

(12) $$\begin{array}{l} a=\left[\left(1+\varepsilon \right)\left(1-\gamma \right)+\left(\xi -1\right){\left(1+\xi \right)}^{2}\right]\times \\ \frac{\left[\left(1+\varepsilon \right)sin\alpha +\left(1-\rho \right)\left(1-\gamma \right)cos^{2}\alpha \right]}{\left[\gamma {\left(1+\varepsilon \right)}^{3}\right]} \end{array}$$

(13) $$\begin{array}{l} b=\left(1-\rho \right)\left(1-\gamma \right)\left[\left(1-\rho \right){\left(1-\gamma \right)}^{2}+\left(\xi -1\right){\left(1+\xi \right)}^{2}\right]\times \\ \frac{\left[1+\xi -\left(1+\varepsilon \right)sin\alpha \right]}{\left[\gamma {\left(1+\xi \right)}^{4}\right]} \end{array}$$

(14) $$c={\left(1-\rho \right)}^{2}\frac{1-\gamma }{1+\xi }$$

Here, γ=φrtanα, and $\rho =1-\bar{r}/r$ represent the variation ratio of the helical radius of the outer wire before and after loading. Using Equation (10), $E_{s}$ can be calculated, and then the strain parameters ε, γ, ξ, ρ are substituted into Equations (12)–(14) to determine a, b, and c. These values are then substituted into Equation (11) to calculate $G_{s}$.

Since ε, γ, ξ, ρ≪1, the third term in the expression for $E_{s}$ is primarily dependent on α. Because α is typically large in the compensation rope, Equation (10) can be further simplified to Equation (15):

(15) $$E_{s}\approx {\left(\frac{d_{0}}{D}\right)}^{2}E_{0}+m{\left(\frac{d}{D}\right)}^{2}Esin\alpha$$

By extending the above formulations to a strand composed of multiple layers of wires, the equivalent elastic modulus of a strand can be expressed by Equation (16):

(16) $$E_{s}\approx {\left(\frac{d_{0}}{D}\right)}^{2}E_{0}+\overset{n}{\underset{i=1}{\sum }}m_{i}{\left(\frac{d_{i}}{D}\right)}^{2}E_{i}sin\alpha_{i}$$

where $E_{s}$ denotes the equivalent elastic modulus of an arbitrary strand in the compensation rope; $d_{0}$ is the diameter of the strand core wire; $E_{0}$ is the elastic modulus of the strand core wire; $d_{i}$ is the diameter of the wires in the i-th layer of the strand; $E_{i}$ is the elastic modulus of the wires in the i-th layer; $\alpha_{i}$ is the helix angle of the centerline of the wires in the i-th layer; $m_{i}$ is the number of wires in the i-th layer; and D is the diameter of the circumscribed circle of the strand.

Accordingly, the equivalent elastic modulus of the entire compensation rope can be expressed by Equation (17):

(17) $$E_{r}\approx {\left(\frac{{d_{0}}^{\prime }}{D^{\prime }}\right)}^{2}{E_{s0}}^{\prime }+\overset{n}{\underset{i=1}{\sum }}{m_{i}}^{\prime }{\left(\frac{{d_{i}}^{\prime }}{D^{\prime }}\right)}^{2}{E_{si}}^{\prime }sin{\alpha_{i}}^{\prime }$$

where $E_{r}$ is the equivalent elastic modulus of the compensation rope; $d_{0}^{\prime }$ is the diameter of the circumscribed circle of the core strand; $E_{s0}^{\prime }$ is the equivalent elastic modulus of the core strand; $d_{i}^{\prime }$ is the diameter of the i-th layer of strands; $E_{si}^{\prime }$ is the equivalent elastic modulus of the i-th layer of strands; $\alpha_{i}^{\prime }$ is the helix angle of the centerline of the strands in the i-th layer; $m_{i}^{\prime }$ is the number of strands in the i-th layer; and $D^{\prime }$ is the diameter of the circumscribed circle of the compensation rope.

Given the equivalent elastic modulus $E_{r}$ of the compensation rope, the axial strain ε can be calculated using Equation (18):

(18) $$\varepsilon =\frac{F}{E_{r}A}$$

where F is the axial tensile force and A is the cross-sectional area of the compensation rope.

If the axial length of the compensation rope is l, the total elongation Δl can be determined by Equation (19):

(19) $$\Delta l=l\varepsilon =\frac{Fl}{EA}$$

### 3.2. Fatigue Life Theory

In engineering practice, the fatigue life of wire ropes is commonly predicted using the material-based Manson–Coffin strain-life equation. This formulation consists of two components: the elastic strain-life term corresponding to high-cycle fatigue and the plastic strain-life term corresponding to low-cycle fatigue. The expression is given in Equation (20):

(20) $$\varepsilon_{a}=\varepsilon_{\mathrm{ea}}+\varepsilon_{pa}=\frac{{\sigma_{f}}^{\prime }}{E}{\left(2N\right)}^{b}+{\varepsilon_{f}}^{\prime }{\left(2N\right)}^{c}$$

Here, $\varepsilon_{a}$ is the total strain amplitude, $\varepsilon_{ea}$ is the elastic strain amplitude, and $\varepsilon_{pa}$ is the plastic strain amplitude. ${\sigma_{f}}^{\prime }$ denotes the fatigue strength coefficient, b is the fatigue strength exponent, ${\varepsilon_{f}}^{\prime }$ is the fatigue ductility coefficient, and c is the fatigue ductility exponent. E represents the elastic modulus, and N is the fatigue life.

In practical service, the compensation rope is predominantly subjected to high-cycle fatigue conditions. Therefore, the fatigue life can be estimated using only the elastic strain-life component. Furthermore, using the relationship between elastic strain amplitude and stress amplitude, $\varepsilon_{ea}=\sigma_{a}/E$, Equation (20) can be simplified to Equation (21).

(21) $$\sigma_{a}={\sigma_{f}}^{\prime }{\left(2N\right)}^{b}$$

Here, $\sigma_{a}$ is the stress amplitude under fully reversed loading, when the stress ratio $R=\sigma_{min}/\sigma_{max}=-1$, under which condition it can be directly substituted into the equation. In the absence of experimental fatigue property data for the steel wire material, the fatigue strength coefficient ${\sigma_{f}}^{\prime }$ can be approximately estimated using the ultimate tensile strength of the steel wire. The corresponding expression is given in Equation (22):

(22) $${\sigma_{f}}^{\prime }=1.75\sigma_{u}$$

where $\sigma_{u}$ denotes the ultimate tensile strength of the steel wire. According to the tensile test results, $\sigma_{u}$ is taken as 2210 MPa. (The tensile strength of the 0.53 mm diameter high-carbon galvanized steel wire shown in Table 2 is 2209.8 MPa.)

The Goodman mean stress correction is applied to account for the effect of the mean stress $\sigma_{m}$, thereby converting the actual service cyclic stress amplitude ${\sigma_{a}}^{\prime }$ into the equivalent stress amplitude $\sigma_{a}$ under fully reversed loading. The corresponding expression is given as follows:

(23) $$\sigma_{m}=\frac{\sigma_{\min}+\sigma_{\max}}{2}$$

(24) $$\frac{{\sigma_{a}}^{\prime }}{\sigma_{a}}+\frac{\sigma_{m}}{\sigma_{u}}=1$$

By substituting the mean stress–corrected stress amplitude $\sigma_{a}$ into Equation (21), the fatigue life of the compensation rope can be calculated.

## 4. Results and Discussion

### 4.1. Structural Configuration of the Compensation Rope

**Figure 10:**
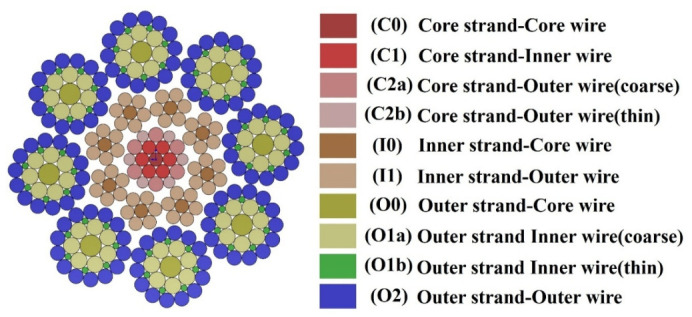
Cross-sectional form of compensation rope.

As shown in the cross-sectional view in Figure 10, the compensation rope consists of one core strand, eight inner-layer strands, and eight outer-layer strands. This cross-sectional configuration corresponds exactly to the actual compensation rope used in the bending fatigue tests described in Section 2.2.3 (nominal diameter 10.9 mm, right-hand alternating lay structure). Each strand is composed of steel wires arranged in multiple layers. For clarity in the subsequent discussion, the core strand, inner-layer strands, and outer-layer strands are denoted by the letters C, I, and O, respectively. The numbers 0, 1, and 2 indicate the different wire layers within a strand from the innermost to the outermost. Within the same layer, thicker and thinner wires are distinguished by the letters a and b, respectively.

The nominal diameter of the compensation rope is 11 mm. The spatial geometric parameters of the three types of strands and their internal steel wires are listed in Table 3. A left-hand lay is denoted by L, and a right-hand lay by R. The inner-layer strands and outer-layer strands share the same lay length and lay direction as the overall rope; however, the lay direction and lay length of the steel wires within each strand differ. The nomenclature of each strand is primarily defined by the lay direction of its outer-layer strands. When the lay direction of the steel wires differs from that of the strand, the structure is defined as a cross-lay. Accordingly, the compensation rope is identified as a right-hand cross-lay wire rope. This cross-lay configuration provides enhanced deformation resistance and load-carrying capacity.

The steel wires within the compensation rope are made of high-carbon galvanized steel, with an elastic modulus E=210 GPa and a Poisson’s ratio ν=0.3. By substituting the structural parameters of the strands and steel wires listed in Table 3 into Equations (16) and (17), the equivalent elastic moduli of the core strand, inner-layer strands, and outer-layer strands are calculated as $E_{sc}=50.67$ GPa, $E_{si}=64.41$ GPa, and $E_{so}=57.95$ GPa, respectively. The overall equivalent elastic modulus of the compensation rope is obtained as $E_{r}=29.76$ GPa.

### 4.2. Establishment of Finite Element Model

**Figure 11:**
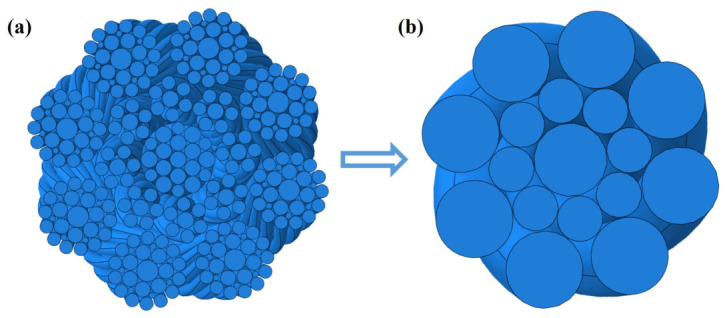
Geometric model of the compensation rope. (**a**) The full-structure model, (**b**) the equivalent diameter simplified model, both of which retain the right-lay structural characteristics of the whole rope and provide a geometric modeling basis for subsequent finite element simulation.

A full-geometry compensation rope model was constructed using SOLIDWORKS software (2023), as shown in Figure 11a. In addition, a simplified geometric model based on the equivalent diameters of the internal strands was developed, in which the individual strand lay direction and lay length were neglected, while the overall rope was assumed to have a constant right-hand lay and lay length, as shown in Figure 11b. The models were saved in Parasolid (*.x_t) format and imported into Abaqus as three-dimensional solid components for nonlinear mechanical analysis.

**Figure 12:**
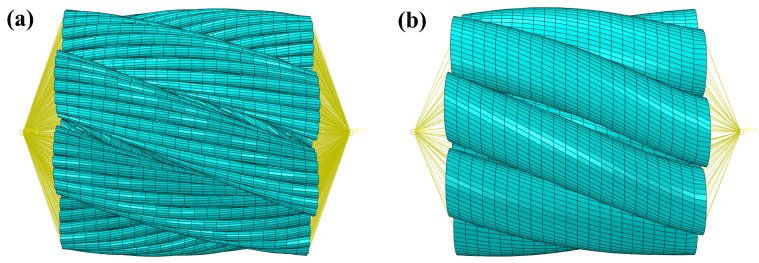
Finite element meshes of the two models. (**a**) Full-structure model. (**b**) Equivalent diameter model.

To verify the validity of the equivalent diameter compensation rope model, two models with the same length of 10 mm were selected, and their overall elongations under an axial tensile load of 15 kN were compared through numerical simulation. As shown in Figure 12, both models were discretized using C3D8R eight-node hexahedral elements, and automatic sweep meshing was performed based on the neutral-axis algorithm. The axial mesh seed size was kept identical for both models. Considering the numerous contacts between individual wires, the general contact algorithm in ABAQUS 2022 was employed, which automatically detects contact interactions during the analysis. In terms of defining contact properties, the tangential behavior adopts the penalty function friction formula, with the friction coefficient set to 0.1. Compared to other friction formulas such as the Lagrangian multiplier method, the penalty function method allows for slight elastic slippage in the tangential direction on the contact surface, effectively improving the computational convergence of complex contact problems. The normal behavior is defined using “Hard Contact”, which establishes strict impermeable constraints between contact surfaces, allowing contact pressure to be transmitted arbitrarily after contact is established. It also permits the separation of contact surfaces when the contact pressure decreases to zero.

To facilitate load application and improve numerical convergence, reference points RP1 and RP2 were established at positions 2 mm away from the centers of the front and rear end faces, respectively, and the corresponding end surfaces were coupled to these reference points. The boundary conditions were defined as follows: RP1 was fully constrained, while RP2 was constrained in axial rotation and subjected to an axial tensile load of 15 kN. The only difference between the two models lay in the definition of material properties. For the full geometric model, the elastic modulus of high-carbon galvanized steel was assigned, whereas for the equivalent diameter model, the core strand, inner-layer strands, and outer-layer strands were assigned the theoretically calculated equivalent elastic moduli $E_{sc}$, $E_{si}$, and $E_{so}$, respectively.

**Figure 13:**
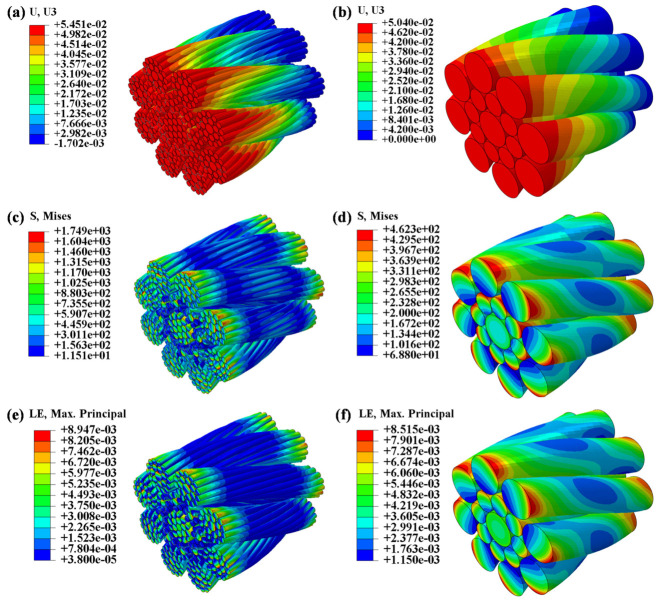
Finite element results for the two models. (**a**) Axial deformation distribution for the full-scale structural model. (**b**) Axial deformation distribution for the equivalent diameter model. (**c**) Stress distribution for the full-scale structural model. (**d**) Stress distribution for the equivalent diameter model. (**e**) Strain distribution for the full-scale structural model. (**f**) Strain distribution for the equivalent diameter model.

To verify the validity of the equivalent diameter model, the axial deformation and the overall stress and strain distributions of the full-structure model and the equivalent diameter model were compared under the same axial tensile load (15 kN), as shown in Figure 13. As shown in the axial deformation distributions in Figure 13a,b, the end elongation of the full-structure model is 54.51 μm, while that of the equivalent diameter model is 50.40 μm. The relative deviation between the two is only 7.5%. Both results agree well with the elongation of 58.1 μm calculated using the theoretical equivalent elastic modulus Er = 29.76 GPa substituted into Equation (19), indicating that the two models exhibit high consistency in their macroscopic stiffness response. As shown in the overall stress distributions in Figure 13c,d, the stresses in both models are uniformly distributed along the axial direction, and their distribution patterns exhibit significant similarity. However, there is a notable difference in stress values: the maximum Mises stress in the complete structural model is approximately 1749 MPa, while that in the equivalent diameter model is approximately 462 MPa. This discrepancy primarily stems from the different physical meanings of the stress outputs in the two models. The high stresses in the full-structure model arise from local contact and squeezing between individual wires and between strands. Even under pure axial tension, the helical winding structure of the wires causes mutual squeezing and normal contact forces between layers of wires, resulting in stress peaks far exceeding the average stress in local contact regions. In contrast, the equivalent diameter model simplifies the complex strand structure into a homogeneous material; the stress it outputs is a macroscopic equivalent stress obtained through volume averaging, and it cannot reproduce the local stress concentrations caused by micro-scale contact. Therefore, the difference in stress magnitude between the two models is an inherent characteristic of the modeling strategies themselves and does not indicate that the equivalent diameter model is invalid. Judging from the overall strain distribution shown in Figure 13e,f, the strain distribution patterns of the two models are essentially consistent. The maximum principal strain for the complete structural model is 8.95 × 10^−3^, while that for the equivalent diameter model is 8.52 × 10^−3^, with a relative error of only 4.8%. This further confirms that the equivalent diameter model possesses high accuracy in predicting macroscopic deformation. Since both the end elongation and macroscopic strain reflect the overall stiffness response of the compensation rope—and this stiffness response is the key parameter determining stress amplitude in subsequent bending fatigue analysis—the equivalent diameter model maintains good consistency with the full-structure model at the level of macroscopic mechanical behavior.

In summary, the comparison of end elongation and strain distribution has fully validated the accuracy of the equivalent diameter model in terms of macroscopic mechanical response, whereas a direct numerical comparison of stress contour plots is not practically comparable due to differences in physical significance. Based on this simplified model, a fatigue life analysis of the compensation rope bending around a ratchet will be conducted in subsequent studies.

**Figure 14:**
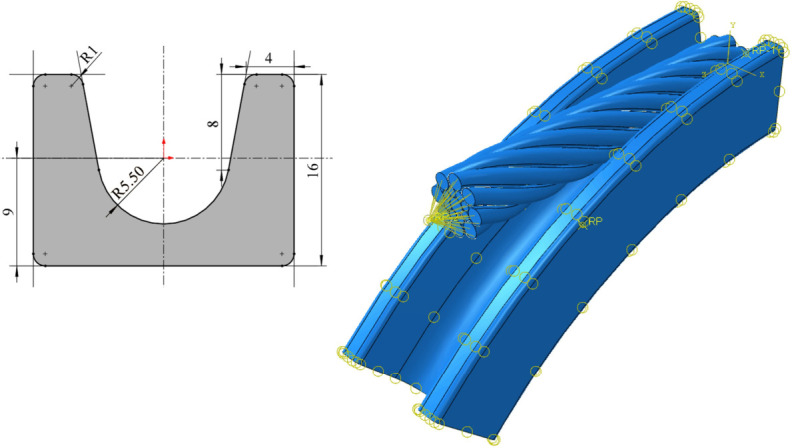
Finite element model of compensation rope bending.

Due to the strong nonlinearities in the full-structure model under bending loading—including large deformation, abrupt changes in contact status, and local buckling—the finite element solver repeatedly encountered convergence failures even after mesh refinement and solver parameter optimization. In contrast, the equivalent diameter model, which simplifies the complex strand structure into a homogeneous material, eliminates these convergence difficulties and enables stable computation. Therefore, an equivalent diameter model with a length of 60 mm and a ratchet model with a diameter of 180 mm represented by a 1/8 sector were assembled, as shown in Figure 14. In this paper, the origin is defined at the center of the ratchet model, with the Y-axis pointing vertically upward, the X-axis extending horizontally to the right, and the Z-axis extending outward perpendicular to the plane of the paper (following the right-hand rule). In finite element analysis, U1, U2, and U3 represent the translational degrees of freedom along the X, Y, and Z axes, respectively, while UR1, UR2, and UR3 represent the rotational degrees of freedom about the X, Y, and Z axes, respectively. The inner radius of the ratchet, R=5.5 mm, effectively restricts lateral oscillation of the compensation rope on its surface and prevents derailment. The ratchet was treated as a rigid body and controlled using a reference point (RP). Similarly, reference points RP1 and RP2 were established at positions 5 mm away from the centers of the front and rear end faces of the compensation rope, respectively, and the corresponding surfaces were coupled to facilitate load application. The bending simulation consisted of three analysis steps. In the first step, a force of −10 N was applied to RP1 in the Y-direction, and this force was removed before the end of the step using an amplitude definition. All degrees of freedom of RP1 and RP2 were fixed except for the U2 direction. The purpose of this step was to initiate contact between the compensation rope and the ratchet. In the second step, all degrees of freedom of RP1 were constrained, and a displacement of −20.5 mm was applied to RP2 in the U2 direction. All other degrees of freedom were fixed except for U3 and UR1. This step aimed to bend the compensation rope and bring it into full contact with the ratchet surface, representing a pure bending loading process. In the third step, all degrees of freedom of RP1 remained constrained, while all degrees of freedom of RP2 were fixed except for U2 and U3. A compressive pressure of −228 MPa was applied to the left end face after bending. This step was intended to simulate the subsequent tensile loading of the compensation rope after bending, corresponding to a coupled tension–bending loading condition.

### 4.3. Finite Element Analysis Results

**Figure 15:**
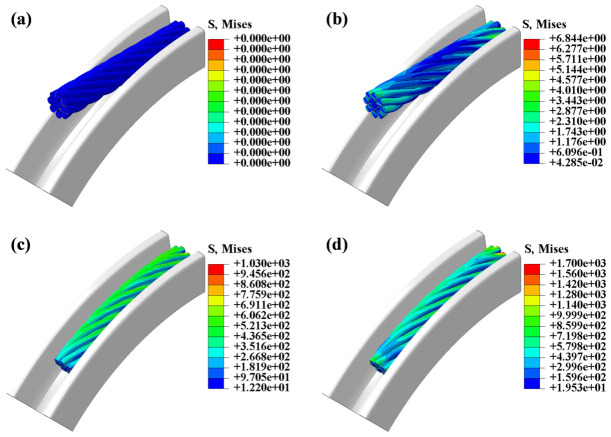
Contour plots of stress distribution at different analysis steps. (**a**) Not. (**b**) Step 1. (**c**) Step 2. (**d**) Step 3.

The stress distributions of the compensation rope under the unloaded condition and after the three loading analysis steps are shown in Figure 15. After the first analysis step, due to contact and squeezing between the right end of the compensation rope and the ratchet, a small residual stress remained after unloading; however, its magnitude was only 6.84 MPa and can be neglected, as shown in Figure 15b. After the second analysis step corresponding to pure bending, the stress distribution on the outer surface of the compensation rope was relatively uniform, and the maximum von Mises stress reached 1030 MPa, as shown in Figure 15c. After the third analysis step involving coupled tensile–bending loading, the stress distribution on the outer surface became non-uniform, with pronounced stress concentrations in the outer-layer strands at the right end; the maximum von Mises stress increased to 1700 MPa, as shown in Figure 15d.

**Figure 16:**
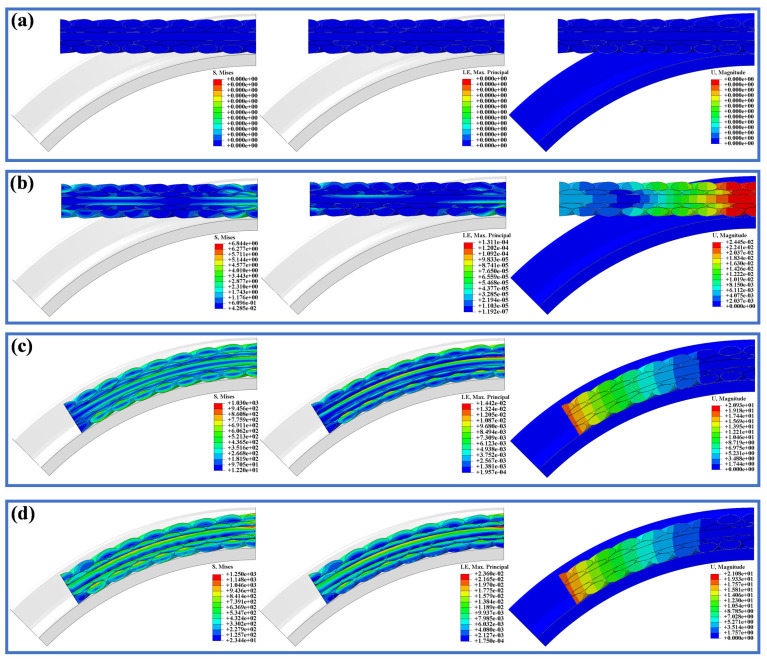
Contour plots of stress, strain, and deformation distributions on cross-sections at different analysis steps. (**a**) Not. (**b**) Step 1. (**c**) Step 2. (**d**) Step 3.

The internal stress–strain and deformation distributions of the compensation rope cross-section after each loading analysis step are shown in Figure 16. In the unloaded state, no stress or deformation was observed in the internal strands. After completion of the first contact loading step, the deformation of the right-end strands was the largest, indicating contact between the compensation rope and the ratchet; only very small residual stresses and strains remained in the strands and can be neglected. After the second pure bending loading step, high-stress bands appeared on the strand surfaces, while low-stress bands developed in the interior, distributed along the inner and outer sides of the bending neutral layer. The maximum stress occurred on the outer bending surfaces of the core strand and the outer-layer strands. High-strain regions were observed on the outer side of bending, whereas low-strain regions appeared on the inner side. The maximum strain 1.442 × 10^−2^ occurred on the outer bending surfaces of the core strand and the outer-layer strands. The left-end strands exhibited relatively larger bending deformation, with a maximum deformation of 20.93 mm. After completion of the third tensile–bending coupled loading step, the strand stresses were no longer uniformly distributed along the bending-neutral layer. Pronounced stress concentration appeared on the outer bending surface of the core strand at the right end, reaching 1250 MPa, and the high-stress regions on the outer bending surfaces of the outer-layer strands further expanded. The strain level increased accordingly; consistent with the pure bending case, the maximum strain increased to 2.36 × 10^−2^ and was located on the outer bending surface of the core strand. Similarly, the maximum deformation still occurred at the left end, reaching 21.08 mm.

**Figure 17:**
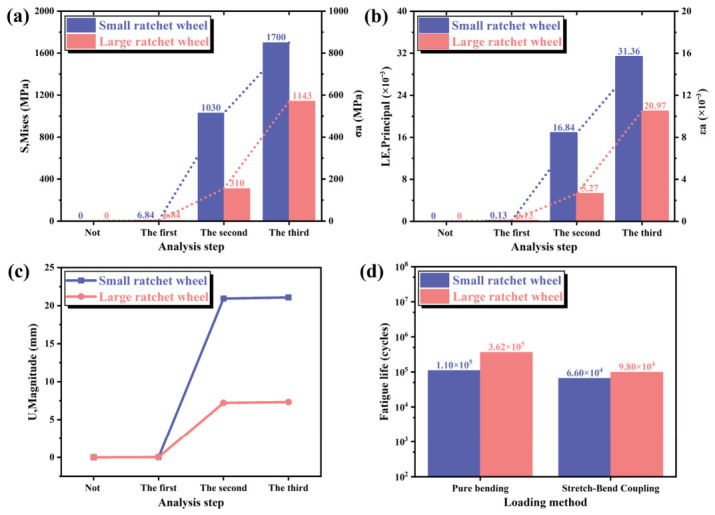
Comparison of compensation rope bending under ratchets with different diameters. (**a**) Stress. (**b**) Strain. (**c**) Deformation. (**d**) Fatigue Life.

The actual compensation device is assembled using a small ratchet with a diameter of 180 mm and a large ratchet with a diameter of 566 mm, as shown in Figure 17, which compares the mechanical performance of the compensation rope under bending over the two ratchets. As illustrated in Figure 17a,b, the left Y-axis represents the stress and strain indicated by the bar charts, while the right Y-axis represents the stress amplitude and strain amplitude indicated by the dashed curves. For the small ratchet (180 mm diameter), under pure bending, the maximum von Mises stress is 1030 MPa and the maximum strain is 1.68 × 10^−2^; under tension–bending coupled loading, these values increase to 1700 MPa and 3.14 × 10^−2^, respectively. For the large ratchet (566 mm diameter), under pure bending, the maximum von Mises stress is 310 MPa and the maximum strain is 5.27 × 10^−2^; under tension–bending coupled loading, these values increase to 1143 MPa and 2.10 × 10^−2^, respectively. Based on these numerical values, it can be observed that the tension–bending coupled loading condition produces significantly higher stress and strain levels than pure bending for both ratchets. Furthermore, the smaller ratchet diameter results in larger bending curvature of the compensation rope, which causes greater tensile strain on the outer side of the neutral layer and greater compressive strain on the inner side, as well as more severe inter-strand squeezing and friction. Consequently, the stress and strain amplitudes for the small ratchet (stress amplitude 850 MPa, strain amplitude 1.57 × 10^−2^) are considerably higher than those for the large ratchet (stress amplitude 572 MPa, strain amplitude 1.05 × 10^−2^), leading to a shorter fatigue life. As shown in Figure 17c, a smaller ratchet diameter corresponds to a larger bending curvature of the compensation rope, resulting in greater overall deformation. The deformation of the compensation rope over the large ratchet is 7.32 mm, whereas it reaches 21.08 mm over the small ratchet. By substituting the obtained stress amplitudes into the fatigue life Equation (21), the fatigue lives of the compensation rope under bending over the two ratchet diameters are calculated. The results are presented in Figure 17d. The predicted fatigue life for the large ratchet under tension–bending coupled loading is 9.8 × 10^4^ cycles, while that for the small ratchet is 6.6 × 10^4^ cycles. The fatigue life of the compensation rope when bent over the large ratchet is higher than that over the small ratchet.

### 4.4. Fatigue Experimental Results

**Figure 18:**
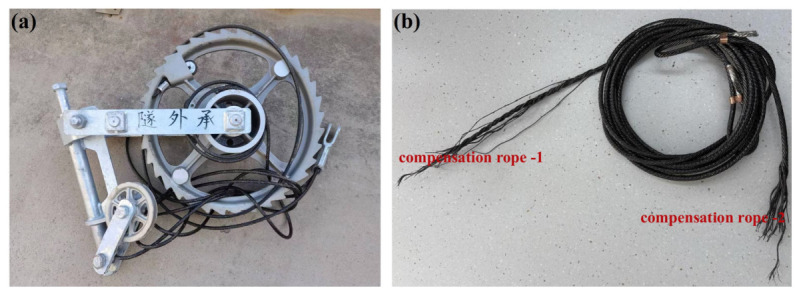
Intact compensation rope specimen without pre-existing damage. (**a**) Before the fatigue test (The Chinese characters mean they are adopted outside of tunnels). (**b**) After the fatigue test.

To determine the fatigue life of the compensation rope under tensile–bending coupled loading during normal service conditions, fatigue tests were conducted on intact specimens with no initial damage in accordance with the test method described in Section 2.2.3. Specifically, the compensation rope was symmetrically wound around two 180 mm diameter grooved pulleys on either side of a ratchet mechanism and suspended with a 30 kN counterweight. A hydraulic cylinder drove the system at a frequency of 3 cycles per minute; each complete up-and-down movement of the counterweight was counted as one fatigue cycle. The test continued until the compensation rope broke, at which point the cumulative number of cycles was recorded as the fatigue life. The specimen before testing is shown in Figure 18a, and after fracture in Figure 18b. The test was terminated at 64,000 cycles due to the compensating rope breaking; therefore, the measured fatigue life is 6.4 × 10^4^ cycles.

**Figure 19:**
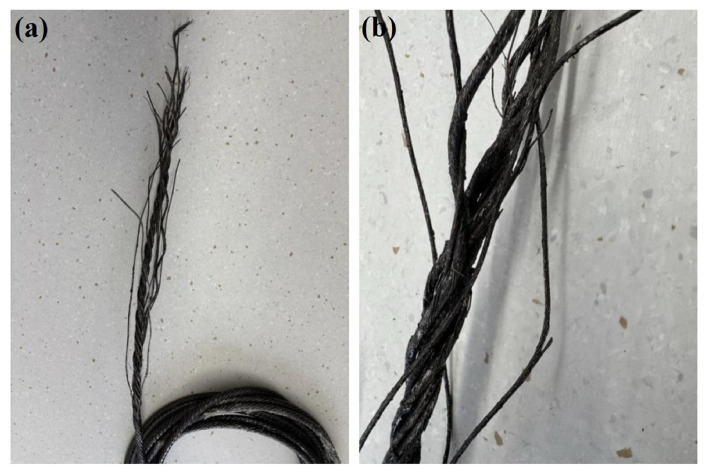
Macroscopic features of the fracture surface of compensation rope 1. (**a**) The overall morphology of the fracture, (**b**) the local magnified detail of the fracture. The fracture consists of one core strand + six inner strands + eight outer strands, with oil stains attached to the surface, and the strands around the fracture position are loose with broken strands of different lengths.

**Figure 20:**
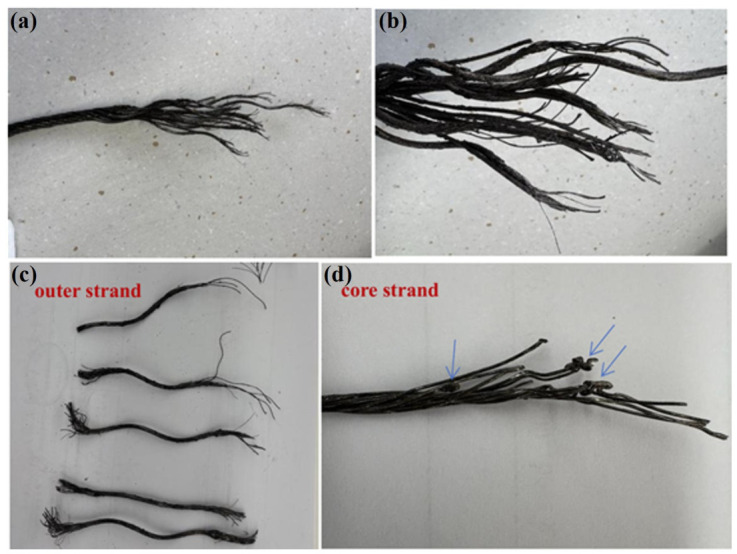
Macroscopic features of the fracture surface of compensation rope 2. (**a**) The overall morphology of the fracture, (**b**) the local magnified detail of the fracture, (**c**) the close-up of the core wire deformation area, and (**d**) the close-up of the outer strand fracture area. The fracture structure is consistent with that of compensation rope 1, and the single wires of the core strand have obvious deformation, which is the key area for micro-morphology observation.

The two fracture sections were designated as compensation rope 1 and compensation rope 2, respectively. As shown in Figure 19 and Figure 20, both fracture sections consist of one core strand, six inner-layer strands, and eight outer-layer strands. The overall fracture surfaces appear black and are covered with a large amount of grease and other contaminants. In the vicinity of the fracture locations, the strands are loosened, and the broken wires exhibit different fracture lengths. Notably, in the core strand region of compensation rope 2, obvious plastic deformation of individual wires is observed, as indicated by the blue arrow in Figure 20d. Therefore, scanning electron microscopy (SEM) was performed to further examine the fracture morphology.

**Figure 21:**
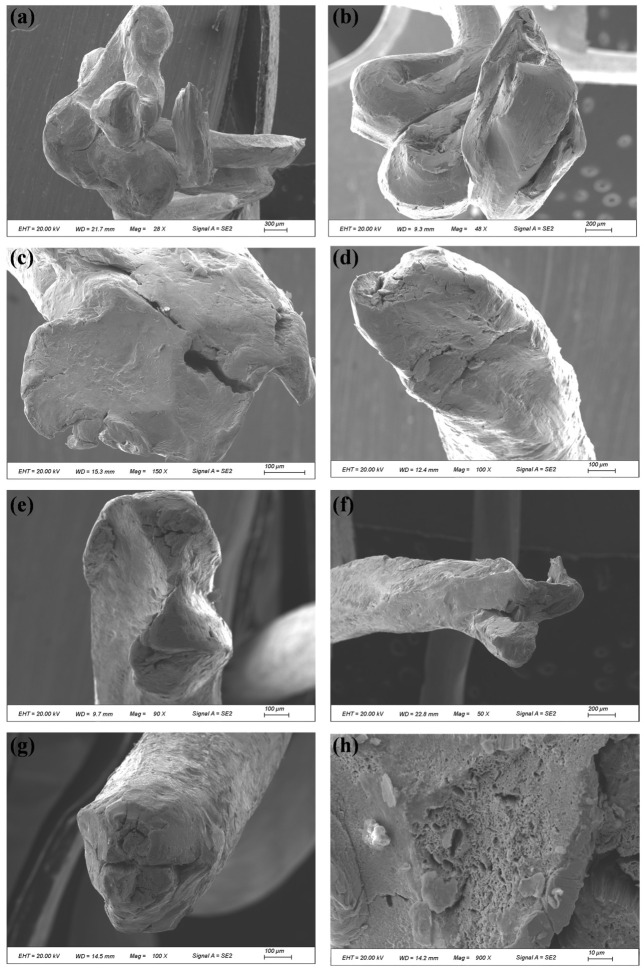
Microscopic morphology of fracture surfaces of different core wires in compensation rope 2. Figures (**a**–**h**) show the fracture characteristics of different single wires in sequence. Most single wires have severe extrusion and wear damage on the surface and fracture, and a small number of single wires show necking characteristics with dimple morphology at high magnification.

During the macroscopic examination of the fracture surface of compensation rope 2, severe bending and plastic deformation of individual wires in the core strand were observed. These wires were subsequently examined by scanning electron microscopy (SEM) to characterize their microscopic morphology. The results are shown in Figure 21. Severe extrusion and wear damage were observed on both the surfaces and fracture sections of most core wires, and the original fracture morphology could not be clearly identified. A limited number of wires exhibited relatively distinct necking features, which presented typical ductile dimple morphology at higher magnification.

**Figure 22:**
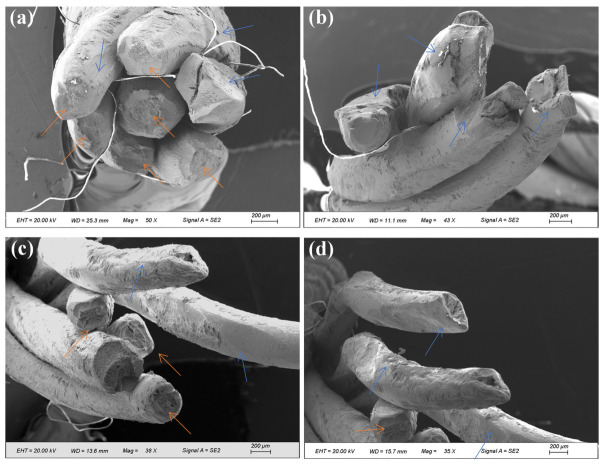
Microscopic morphology of the inner-layer strand at the fracture section of compensation rope 2. (**a**–**d**) Single wire morphology of the inner strand from different perspectives/positions. The blue arrows mark the areas with severe extrusion wear, and the orange arrows mark the typical necking characteristics of the single-wire fracture surface, showing the failure characteristics of coexistent wear and plastic deformation as a whole.

**Figure 23:**
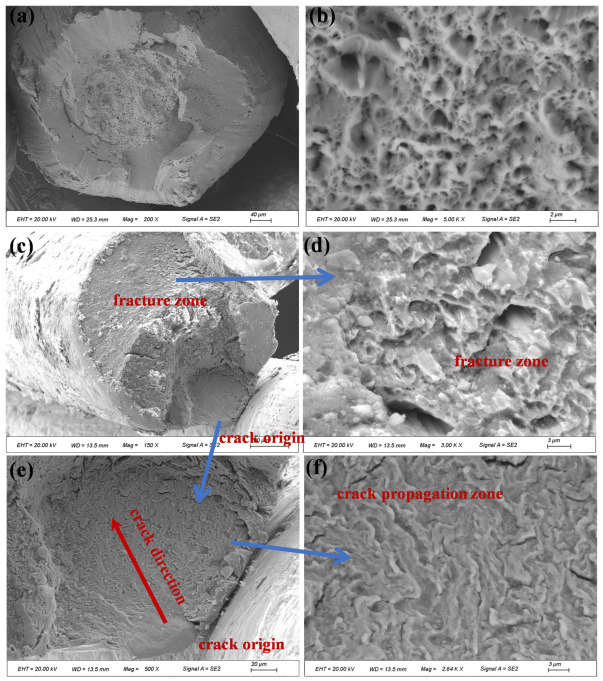
Microscopic morphology of fracture surfaces of different inner-layer wires in compensation rope 2. (**a**–**f**) The crack characteristics and fracture morphology of different single wires. Single-source cracks propagating from the surface to the inside can be seen, with extrusion wear marks at the crack source, no obvious fatigue striations, and the instantaneous fracture zone is all of dimple morphology.

Figure 22 and Figure 23 present the microscopic morphology of different wires in the inner-layer strand at the fracture section of compensation rope 2. Severe extrusion and wear damage were observed on the surfaces, fracture sections, and surrounding areas of some wires, as indicated by the blue arrows in Figure 22, making it difficult to identify their original fracture characteristics. Most wire fractures exhibited typical necking features (indicated by the orange arrows in Figure 22), and ductile dimple morphology was observed at higher magnification (Figure 23). For certain wires, a single crack initiation site was identified (Figure 23). The crack initiated at the wire surface and propagated inward. No distinct fatigue striations were observed at higher magnification. However, evident extrusion and wear damage traces were present at the crack initiation location. The final rapid fracture zone exhibited typical ductile dimple morphology.

**Figure 24:**
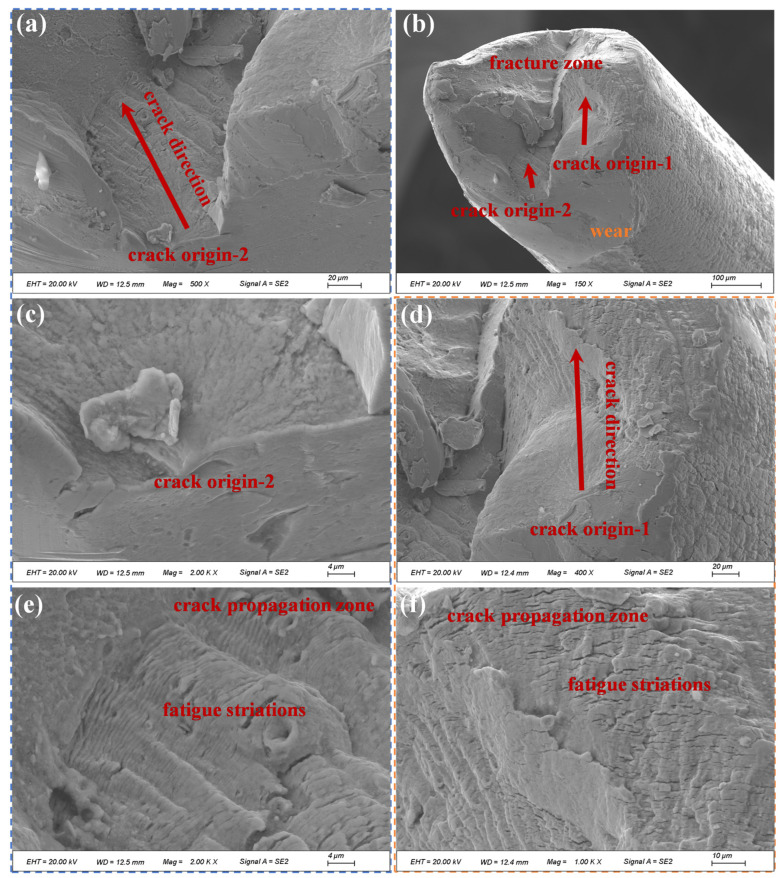
Microscopic morphology of fracture surfaces of outer-layer wires in compensation rope 2. (**a**–**f**) Fatigue fracture characteristics of single wires in different fields of view. Multi-source cracks all originate from the single wire surface and propagate inward, with obvious fatigue striations in the propagation zone, extrusion wear marks at the crack source, and dimple morphology in the instantaneous fracture zone.

**Figure 25:**
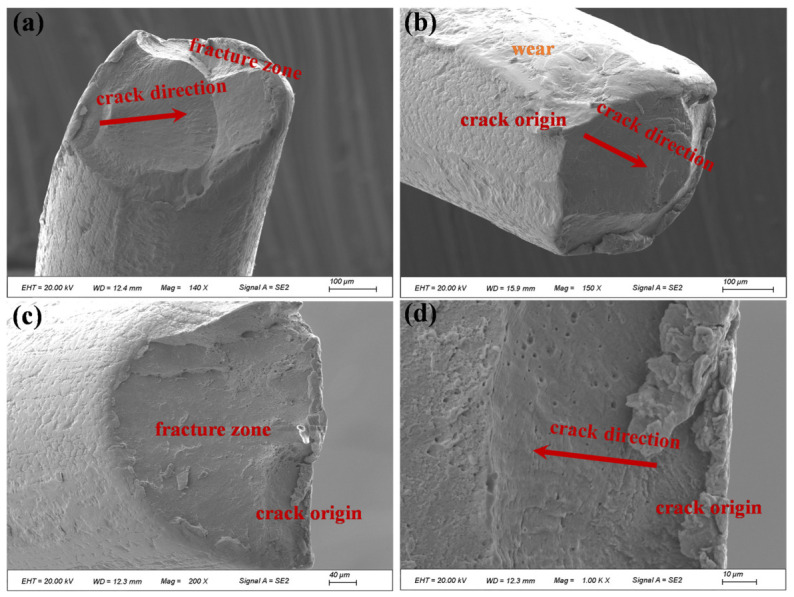
Microscopic morphology of fracture surfaces of different outer-layer wires in compensation rope 2. (**a**–**d**) Fracture characteristics of different single wires, all showing single-source fatigue fracture attributes. Cracks propagate from the surface to the inside, with wear marks in the source area, fatigue striations in the propagation zone, and dimple morphology in the instantaneous fracture zone.

**Figure 26:**
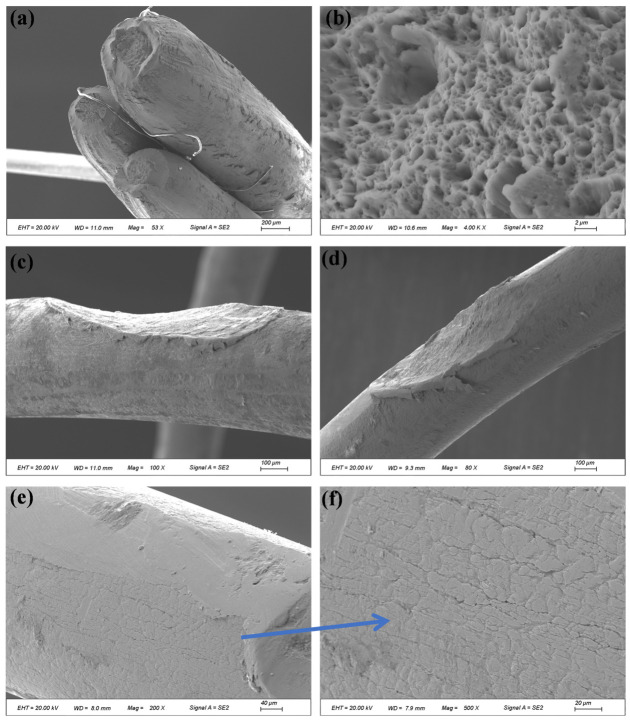
Microscopic morphology of selected outer-layer wires in compensation rope 2. (**a**–**f**) The surface and fracture characteristics of different single wires. Some single-wire fractures have necking and dimple characteristics, some single-wire surfaces have extrusion wear and bump damage, and some single wires retain rough processing marks, reflecting the coexistence of multiple types of damage.

Figure 24, Figure 25 and Figure 26 present the microscopic morphology of fracture surfaces of different individual wires in the outer-layer strand at the fracture section of compensation rope 2. Multiple-crack initiation sites (Figure 24) and single-crack initiation sites (Figure 25) were observed on certain wire fracture surfaces. In all cases, cracks initiated at the wire surface and propagated inward. Distinct fatigue striations were observed during crack propagation, which are characteristic features of fatigue fracture. Clear traces of extrusion and wear damage were identified at the crack initiation sites. The final rapid fracture zone exhibited typical ductile dimple morphology. As shown in Figure 26, some wire fractures displayed pronounced necking features, and typical ductile dimple morphology was observed at higher magnification. In addition, obvious extrusion wear and impact damage marks were present on the surfaces of certain wires, whereas other wires exhibited relatively rough surfaces with evident machining marks.

The bending fatigue test results indicate that no evident corrosion traces were observed on the fracture surfaces of the compensation rope, thereby excluding corrosion as a contributing factor. Fatigue fracture characteristics, including multi-source crack initiation, were identified in several outer-layer wires. All cracks initiated from the wire surface and propagated inward. Extrusion and wear damage were consistently observed at the crack initiation regions, and no other abnormal defects were detected. At higher magnification, distinct fatigue striations were visible in the crack propagation zone, confirming typical fatigue crack growth behavior, while the final fracture zone exhibited ductile dimple morphology. These observations demonstrate that the failure mode of the compensation rope is fatigue fracture.

## 5. Conclusions

This paper proposes an equivalent diameter model for compensation ropes based on Love’s theory of elastic slender rods. By combining finite element analysis with a stress-based method for predicting bending fatigue life, the fatigue behavior of compensation ropes under repeated bending caused by a ratchet mechanism is investigated. It should be noted that, to ensure computational feasibility and numerical convergence, this study employs a series of idealized assumptions, including homogeneous and isotropic material properties, the neglect of initial defects, idealized geometric structures, and the disregard of material degradation during service. These assumptions may lead to an underestimation of local stress concentrations and damage accumulation, resulting in overly optimistic predictions of bending fatigue life. Under these conditions, the following conclusions are drawn:

(1) Based on Love’s elastic slender rod theory, the equivalent elastic moduli of the core strand, inner-layer strand, outer-layer strand, and the compensation rope were calculated to be 50.67 GPa, 64.41 GPa, 57.95 GPa, and 29.76 GPa, respectively. In comparison, the elastic moduli obtained from tensile tests by fitting the stress–strain curves of the corresponding strands were 44.44 GPa, 62.5 GPa, 50.51 GPa, and 25.1 GPa. The observed discrepancies are primarily attributed to the non-uniform deformation of the compensation rope under actual loading conditions, which does not strictly satisfy the assumption of uniform deformation across the cross-section. This behavior is related to inter-wire friction; however, its effect is minimal under well-lubricated conditions.

(2) An equivalent diameter model was adopted as a simplified representation of the full structural model of the compensation rope. Under identical axial tensile loads within the elastic range, the end elongations predicted by the equivalent diameter model, the full-structure finite element model, and the theoretical calculation based on Love‘s elastic slender rod theory are in close agreement with each other.

(3) The stress amplitudes obtained from finite element analysis of the compensation rope bent over ratchets with two different diameters were substituted into the stress-based fatigue life equation. Under repeated pure bending, the predicted fatigue life of the compensation rope over the large ratchet is 3.62 × 10^5^ cycles, whereas it decreases to 1.1 × 10^5^ cycles over the small ratchet. Under repeated coupled tension–bending loading, the fatigue life over the large ratchet is 9.8 × 10^4^ cycles, while that over the small ratchet is 6.6 × 10^4^ cycles (see Section 4.3, Figure 17d). The predicted fatigue life for the small ratchet shows good agreement with the experimentally measured value of 6.4 × 10^4^ cycles (see Section 4.4).

## Figures and Tables

**Table 1:** Chemical Composition of materials (wt. %).

Steel Wire Material	C	Si	Mn	P	S	Cr	Ni
High-carbon gal-vanized	0.69	0.22	0.71	0.0086	0.0051	/	/
Stainless	0.023	0.36	1.04	0.0032	0.0009	18.11	8.00

**Table 2:** Test results of steel wires with different diameters.

Steel Wire Material	Diameter /mm	Sectional Area /mm^2^	Sample	Breaking Force /N	Tensile Strength /MPa	Average Value /MPa
Stainless	0.46	0.1661	1/2/3/4	316/322/321/321	1862/1897/1892/1892	1885.8
High-carbon galvanized	0.20	0.0314	1/2/3/4	72/68/69/68	2293/2166/2197/2166	2205.5
	0.43	0.1451	1/2/3/4	319/315/312/321	2197/2171/2149/2209	2181.5
	0.47	0.1734	1/2/3/4	372/373/366/369	2145/2151/2111/2128	2133.8
	0.53	0.2205	1/2/3/4	490/482/488/489	2222/2186/2213/2218	2209.8
	0.55	0.2375	1/2/3/4	492/489/494/494	2070/2057/2079/2078	2071.0
	0.57	0.2550	1/2/3/4	514/523/531/532	2016/2051/2082/2086	2058.8
	0.74	0.4299	1/2/3/4	796/788/796/776	1851/1833/1850/1803	1834.3

**Table 3:** Geometric structural parameters of the compensation rope.

Strand Layer	Strand Diameter /mm	Strand Pitch /mm	Strand Helical Radius/mm	Wire Layer	Wire Diameter /mm	Wire Pitch /mm	Wire Helical Radius/mm
Core strand	2.55	0	0	C0	0.57	0	0
				C1	0.55	20(L)	0.57
				C2a	0.57	20(L)	0.98
				C2b	0.43	20(L)	1.06
Inner strand	1.57	60(R)	2.07	I0	0.57	0	0
				I1	0.47	10(R)	0.64
Outer strand	2.68	60(R)	3.91	O0	0.74	0	0
				O1a	0.53	20(L)	0.64
				O1b	0.20	20(L)	0.85
				O2	0.46	20(L)	1.11

## Data Availability

The original contributions presented in this study are included in the article. Further inquiries can be directed to the corresponding authors.
